# The effect of treatment and clinical course during Emergency Department stay on severity scoring and predicted mortality risk in Intensive Care patients

**DOI:** 10.1186/s13054-022-03986-2

**Published:** 2022-04-19

**Authors:** Bart G. J. Candel, Wouter Raven, Heleen Lameijer, Wendy A. M. H. Thijssen, Fabian Temorshuizen, Christiaan Boerma, Nicolette F. de Keizer, Evert de Jonge, Bas de Groot

**Affiliations:** 1 Department of Emergency Medicine, Leiden University Medical Centre, Albinusdreef 2, 2300 RC Leiden, The Netherlands; 2 Department of Emergency Medicine, Máxima Medical Centre, De Run 4600, 5504 DB Veldhoven, The Netherlands; 3 Department of Emergency Medicine, Medical Centre Leeuwarden, Henri Dunantweg 2, 8934 AD Leeuwarden, The Netherlands; 4 Department of Emergency Medicine, Catharina Hospital Eindhoven, Michelangelolaan 2, 5623 EJ Eindhoven, The Netherlands; 5 Department of Medical Informatics, Amsterdam University Medical Center, Meibergdreef 9, 1105 AZ Amsterdam, The Netherlands; 6 Amsterdam Public Health, Quality of Care, Amsterdam, The Netherlands; 7 National Intensive Care Evaluation (NICE) Foundation, Amsterdam, The Netherlands; 8 Department of Intensive Care, Medical Center Leeuwarden, Henri Dunantweg 2, 8934 AD Leeuwarden, The Netherlands; 9 Department of Intensive Care Medicine, Leiden University Medical Centre, Albinusdreef 2, 2300 RC Leiden, The Netherlands

**Keywords:** APACHE III, Data quality, Intensive care, Benchmarking, Medical registries

## Abstract

**Background:**

Treatment and the clinical course during Emergency Department (ED) stay before Intensive Care Unit (ICU) admission may affect predicted mortality risk calculated by the Acute Physiology and Chronic Health Evaluation (APACHE)-IV, causing lead-time bias. As a result, comparing standardized mortality ratios (SMRs) among hospitals may be difficult if they differ in the location where initial stabilization takes place. The aim of this study was to assess to what extent predicted mortality risk would be affected if the APACHE-IV score was recalculated with the initial physiological variables from the ED. Secondly, to evaluate whether ED Length of Stay (LOS) was associated with a change (delta) in these APACHE-IV scores.

**Methods:**

An observational multicenter cohort study including ICU patients admitted from the ED. Data from two Dutch quality registries were linked: the Netherlands Emergency department Evaluation Database (NEED) and the National Intensive Care Evaluation (NICE) registry. The ICU APACHE-IV, predicted mortality, and SMR based on data of the first 24 h of ICU admission were compared with an ED APACHE-IV model, using the most deviating physiological variables from the ED or ICU.

**Results:**

A total of 1398 patients were included. The predicted mortality from the ICU APACHE-IV (median 0.10; IQR 0.03–0.30) was significantly lower compared to the ED APACHE-IV model (median 0.13; 0.04–0.36; *p* < 0.01). The SMR changed from 0.63 (95%CI 0.54–0.72) to 0.55 (95%CI 0.47–0.63) based on ED APACHE-IV. Predicted mortality risk changed more than 5% in 321 (23.2%) patients by using the ED APACHE-IV. ED LOS > 3.9 h was associated with a slight increase in delta APACHE-IV of 1.6 (95% CI 0.4–2.8) compared to ED LOS < 1.7 h.

**Conclusion:**

Predicted mortality risks and SMRs calculated by the APACHE IV scores are not directly comparable in patients admitted from the ED if hospitals differ in their policy to stabilize patients in the ED before ICU admission. Future research should focus on developing models to adjust for these differences.

**Supplementary Information:**

The online version contains supplementary material available at 10.1186/s13054-022-03986-2.

## Background

The Acute Physiology and Chronic Health Evaluation (APACHE)-IV is a scoring system used for risk stratification of Intensive Care Unit (ICU)-patients [1, 2]. The APACHE-IV is also used for case-mix correction when comparing outcomes of patients in national and international research or quality improvement projects.

The acute physiology score (APS), part of the APACHE-IV model, uses the most deviated vital signs and laboratory results measured in the first 24 h after ICU admission [1, 3]. However, physiological parameters may improve or deteriorate in the hours before ICU admission [4–7], e.g., during initial treatment in the emergency department (ED) or because of deterioration in the clinical course. These data measured prior to ICU admission are not included in the original APACHE-IV scores [1, 3]. Consequently, the calculated severity of illness and predicted mortality risk may differ when for a given patient the same initial treatment is given before or after ICU admission or if the ED stay is shorter resulting in a different clinical course, a phenomenon called lead-time bias in the literature [8, 9]. For appropriate risk stratification and case-mix correction, it is essential to understand whether there is an effect of ED treatment on the APACHE-IV scores.

The present study assessed if, and to what extent, the APACHE-IV score and predicted mortality risk change if the APACHE-IV is recalculated, including data from both the pre-ICU ED period and the first 24 h of ICU treatment. In this way we evaluated if earlier findings from a very small sample could be confirmed [9]. Our second aim was to assess whether ED treatments (e.g., the amount of fluid administered) and clinical course during ED stay were associated with changes in the APACHE-IV score, predicted mortality risk, and standardized mortality ratio (SMR).

## Methods

### Study design and setting

This was an observational multicenter cohort study using the Netherlands Emergency department Evaluation Database ((NEED), http://www.stichting-need.nl) and the National Intensive Care Evaluation (NICE) registry [10]. Data were available from two urban teaching hospitals (Medical Center Leeuwarden, 14 October 2017–31 July 2020 and Catharina Hospital Eindhoven, 01 January 2019–01 July 2020). In both hospitals, patients were treated and stabilized in the ED before ICU admission by emergency physicians with or without consultation of an intensivist. If necessary, mechanical ventilation was started in the ED. Patients were almost never intubated prehospital because hospitals can be reached within 15 min by ambulance in both regions in the Netherlands. The medical ethical committee of Máxima MC reviewed the research proposal and concluded that the anonymized data were not subject to the Dutch Research on Humans Subjects Act (in Dutch “WMO”) and waived the need for informed consent (registration number N20.117).

### Patient selection

All ICU patients who were directly admitted from the ED in the mentioned study period were included. Patients transferred to other hospitals and patients without any registered vital signs or laboratory results (9 patients) in the NEED were excluded.

### Data collection

#### Data from the NICE

The NICE is the national quality registry founded in 1996 in which all ICUs in the Netherlands have participated since 2016 [11]. Data available from NICE were: Age, sex, date of admission, ICU length of stay, ICU mortality, hospital mortality, and all data to calculate the APACHE-IV score [1, 12]. Additional file 1 shows which variables were extracted from the NICE and from the NEED to calculate the APACHE-IV score. Only the lowest and the highest values of vital signs and laboratory tests in the first 24 h of ICU admission were registered in the NICE. The NICE registry uses strict definitions and specifications of the data collected, described in a data dictionary (https://www.stichting-nice.nl/dd/). The NICE provides participants a mandatory training and performs automated checks on data entry and regular on-site quality audits [11, 13].

#### Data from the NEED

The NEED is the quality registry for EDs in the Netherlands (http://www.stichting-need.nl), founded in 2016. For the present study, data were available from two hospitals. Only the first set of vital signs and laboratory results were registered in the NEED, measured before ED treatment, described in detail previously [14]. Except for variables to calculate the APACHE-IV score (Additional file 1), patient characteristics (e.g., triage category according to the Dutch Triage System or Manchester Triage Standard, presenting complaints, diagnostics (Y/N), fluid administration), and ED- length of stay (LOS) were extracted.

#### Linking of NEED and NICE databases

Both NEED and NICE have unique identifying patient numbers that are not identical. In the NICE, patients were selected who were admitted from the ED. In the NEED, patients were selected who were admitted to a high dependency care unit. If the following variables were identical between both databases, patient variables were linked: Date of ED admission (in the NEED) and the date of ICU admission (in the NICE), hospital location, sex, and age of the patient. In addition, for records in NICE with ED as resource but without linkage to a NEED record, the date of ED admission could be one day earlier than the date of ICU admission. These patients were linked by changing the data of ED presentation to one day earlier.

### Outcome measures

Outcome measures were the original ICU APACHE-IV score (Formally the APACHE III score used in the APACHE IV model [1, 3]) and predicted mortality using the most deviated physiological variables from the first 24 h after ICU admission, and the ED APACHE-IV score using the most deviated values from ED admission until 24 h after ICU admission. The SMR is the ratio between the observed number of deaths and the expected deaths in the ICU using the original (ICU model) or modified (ED model) predicted mortality risks. The delta APACHE-IV was calculated as the ED APACHE-IV score–ICU APACHE-IV score.

### Data and data analyses

#### Descriptive statistics

Data are presented as mean with standard deviation (SD) if normally distributed and as median with interquartile range (IQR) if skewed. Categorical data are presented as frequencies (%).

#### Main statistical analyses

In our study, the ICU APACHE-IV score was compared with a recalculated APACHE IV score, called the ED APACHE-IV. According to the original model, the ICU APACHE-IV score was calculated with the most deviating vital signs or blood tests in the first 24 h of ICU admission [3]. Points were assigned for the value that was furthest from a reference value. For example, for heart rate the reference value was 75 bpm. Reference values for all other variables are shown in Additional file 2. According to the APACHE IV definitions [3], more deviating vital signs or laboratory results do not necessarily result in a higher score (see Additional file 2). Based on rules of the original APACHE-IV model, missing physiological variables were considered normal. The ED APACHE-IV included the most deviating vital signs and blood tests from ED admission until 24 h after ICU admission. The data available from the NEED registry to calculate the ED APACHE-IV are described in Additional file 1. The acute physiological variables were recalculated, all other variables in the ED APACHE-IV remained identical to the ICU APACHE-IV (e.g., the Chronic Health Conditions). Urine output and mechanical ventilation (Y/N) were not registered in the NEED. Patients were considered not to be intubated at ED arrival. In the ED, venous blood gas is often obtained instead of an arterial blood gas. If no arterial blood gas was available, the pH from the venous blood gas analysis was used with a correction of 0.03, and the pCO2 was used with a correction of − 4.8 mmHg [15]. PaO2 is not well correlated between venous and arterial blood gas and was therefore considered missing if not available as arterial value. Predicted mortality risks were calculated for the ED APACHE-IV using the Beta’s of the ICU APACHE-IV logistic model. The APACHE-IV scores, Acute Physiology Score (APS), and predicted mortality risks were compared between the ICU and ED model with a Wilcoxon Signed Rank test. In addition, we assessed the discriminative performance of both the ICU and ED APACHE-IV model using a receiver operator characteristic (ROC) curve with area under the curve (AUC) analysis and in-hospital mortality as outcome. Calibration plots for both models were presented.

For our second aim, delta APACHE-IV was calculated (e.g., ED APACHE-IV–ICU APACHE-IV). Delta APACHE-IV was normally distributed, and therefore, univariable and multivariable linear regression analyses were performed to assess the association between ED LOS, fluid administration (0 ml, 0-500 ml, > 500 ml) as independent variables, and a delta APACHE-IV as dependent variable. ED-LOS was divided into quartiles (0–1.7 h, 1.7–2.7 h, 2.7–3.9 h, > 3.9 h). Because of a strong correlation between ED LOS and fluid administration, separate models were used. The following potential confounders were included in both multivariable models: Age, gender, hospital, and the ED APACHE-IV score. Although age is one of the variables included in the APACHE-IV, age may still be an independent predictor for delta APACHE-IV. Fluid administration and ED LOS were included as dummy variables to overcome nonlinear associations. The unstandardized coefficients (Beta's) were presented with 95% Confidence Intervals (95%-CI).

To study whether the ED APACHE-IV was significantly different per quartile ED-LOS, a Kruskal Wallis Test was used.

A *p* value < 0.05 was considered statistically significant. SPSS version 25.0 was used for all data analyses.

## Results

### Patient characteristics

**Fig. 1:**
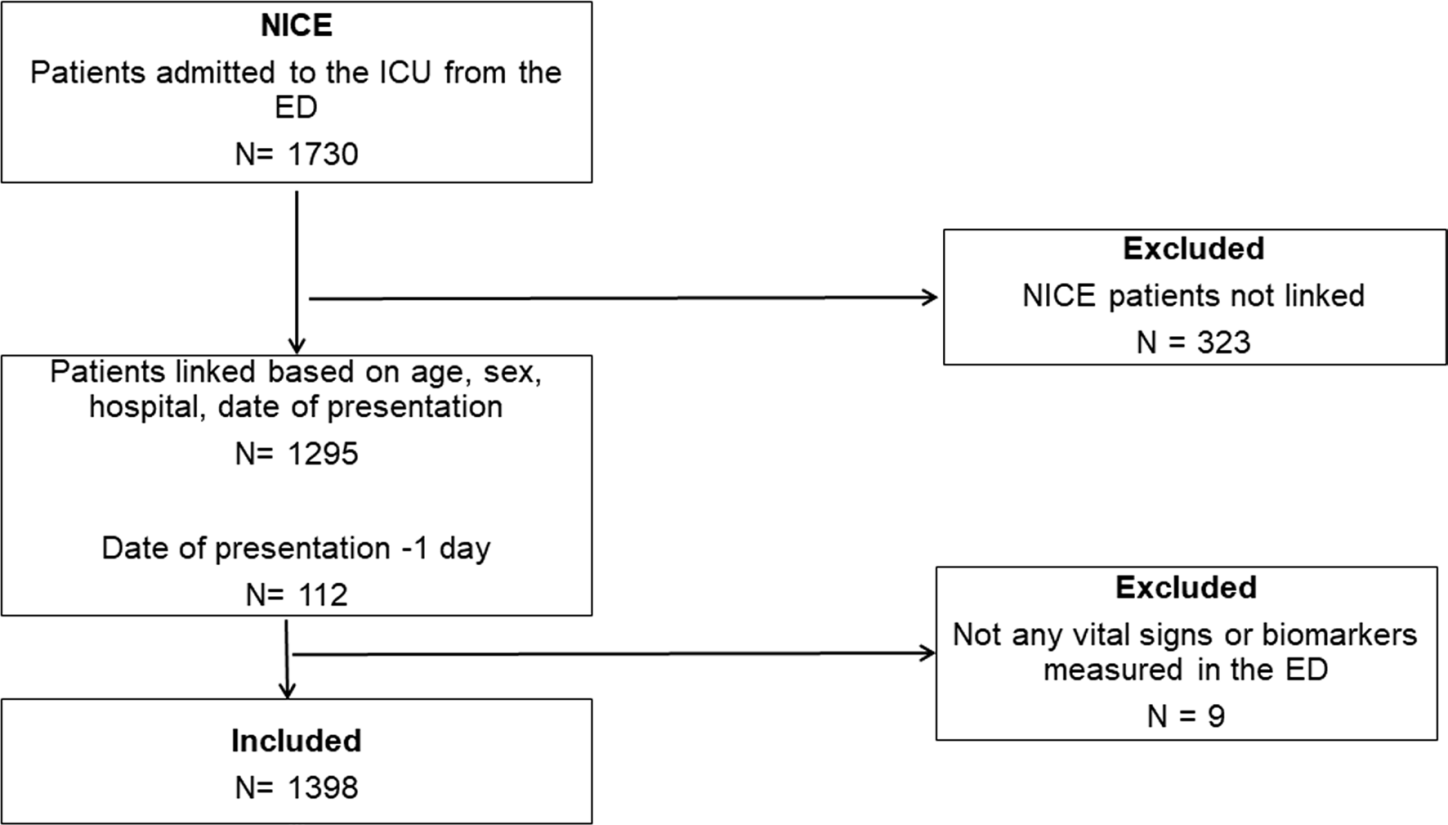
Patient flow diagram throughout the study. Patients from the National Intensive Care Evaluation (NICE) registry were linked with patients from the Netherlands Emergency department Evaluation Database (NEED)

During the study period, 1730 patients were admitted from the ED to the ICU. In total, 1398 (80.8%) patients were included after linking both databases (see Fig. 1). The median age was 64 years (50-74 years), a total of 1046 (60.5%) were male patients. Patient characteristics, including all APS variables, are described in Table 1. Characteristics of patients who could not be linked between both databases (*N* = 323) are comparable with the included patients and are described in Additional file 3. The median laboratory values in the ED were comparable with the lowest and highest values from the ICU. Additional file 4 describes other patient characteristics, such as the triage category in the ED, and the most common presenting complaints in the ED and reasons for ICU admissions.

**Table 1:** Patient characteristics

Characteristics (N = 1398)	Emergency Department	Intensive Care Unit	Intensive Care Unit
Age, years, median (IQR)	64 (50–74)	–	–
Sex, male, N (%)	1046 (60.5)	–	–
*Vital signs, median (IQR) {missing}*	Initial values	Lowest values < 24 h	Highest values < 24 h
MAP, mmHg	97 (80–114) {159}	62 (53–71) {6}	100 (88–115) {4}
HR, bpm	95 (80–114) {156}	71 (59–83) {3}	103 (88–119) {1}
SpO2, %	97 (94–100) {170}	-	-
RR, /min	20 (16–25) {223}	13 (10–16) {8}	25 (22–29) {10}
Temperature, °C	36.7 (36.1–37.3) {429}	36.4 (35.8–36.9) {12}	37.3 (36.8–37.9) {18}
GCS	15 (11–15) {962}	15 (10–15)	
Urine, 24 h, L		1.3 (0.9–2.0)	
*Biomarkers, median (IQR) {missing}*			
Creatinine, µmol/L	88 (71–120) {77}	81 (64–116) {156}	87 (68–128) {164}
Urea, mmol/L	6.5 (4.7–9.4) {76}	7.2 (5.0–10.5) {167}	
Hematocrit, L/L	0.42 (0.37–0.46) {52}	0.36 (0.31–0.40 {115}	0.39 (0.34–0.43) {122}
Leukocytes, × 10^9/L	11.6 (8.2–16.2) {79}	10.9 (7.8–15.0) {225}	12.3 (8.8–17.4) {229}
Sodium, mmol/L	139 (136–142) {53}	137 (134–140) {118}	140 (137–143) {125}
Albumin, g/L	42 (37–45) {828}	29 (24–34) {583}	30 (24–34) {585}
Glucose, mmol/L	7.8 (6.3–11.3) {824}	6.0 (5.2–7.2) {116}	8.8 (7.0–11.6) {124}
Bilirubin, µmol/L	10 (6–15) {431}	8.9 (5.5–14) {547}	
Blood gas, median (IQR) {missing}			
a-PO2, mmHg	86 (60–145) {687}	82 (70–102) {379}	
a/v-PCO2, mmHg	40 (35–50) {346}	39 (33–45) {379}	
a/v-pH	7.36 (7.27–7.43) {344}	7.40 (7.30–7.41) {375}	
*Mechanical ventilation, N (%)*	-	-	611 (43.7)
FiO2 (%), median (IQR)	-	-	35 (25–45) {224}

ED: Emergency Department, ICU: Intensive Care Unit, IQR: Interquartile Range, N: number, MAP: mean arterial pressure, mmHg: millimeter mercury, HR: heart rate, SpO2: peripheral oxygen saturation, RR: respiratory rate, °C: degrees Celsius, GCS: Glasgow Coma Scale, L: Liter, a-PO2: arterial partial pressure of oxygen, a/v-PCO2: arterial or venous partial pressure of carbon dioxide, a/v-pH: arterial or venous acid base, FiO2: Fraction of inspired oxygen

The initial values from the ED are presented before ED treatment. From the ICU, both the lowest and highest values are given from the first 24 h after admission, if available

If arterial blood gas analysis was not available in the ED, venous PCO2 was used with a correction of -4.8 mmHg, and pH was used with a correction of 0.03

### The ED APACHE-IV score

Including the most deviating vital signs and blood tests from ED admission until 24 h after ICU admission, the median ED APACHE-IV score (63; IQR 47–90) was calculated and differed significantly from the median ICU APACHE-IV score (56; IQR 39–80) (*p* value < 0.01). The median predicted mortality for the total population was higher for the ED APACHE-IV system, 0.13 (IQR 0.04–0.36) versus 0.10 (IQR 0.03–0.30) (see Table 2). Observed in-hospital mortality was 13.4% (*N* = 188). The SMR decreased from 0.63 (95% CI 0.54–0.72) for the ICU APACHE-IV model to 0.55 (95% CI 0.47–0.63) for the ED APACHE-IV model, a relative difference of 12.7%.

**Table 2:** The ICU and ED APACHE-IV model

Risk scores	ICU APACHE-IV model	ED APACHE-IV model	*p* value
APACHE-IV score, median (IQR)	56 (39–80)	63 (47–90)	< 0.01
APS, median (IQR)	44 (30–68)	52 (35–76)	< 0.01
APACHE-IV Predicted mortality, median (IQR)	0.10 (0.03–0.30)	0.13 (0.04–0.36)	< 0.01
SMR (95% CI)	0.63 (0.54–0.72)	0.55 (0.57–0.63)	

ICU: Intensive Care Unit, ED: Emergency Department, APACHE-IV: Acute Physiology and Chronic Health Evaluation (4^th^ edition), APS: Acute Physiology Score, SMR: Standardized mortality Ratio (observed mortality / predicted mortality). IQR: interquartile range, 95% CI: 95 percent Confidence Interval

The ED APACHE-IV was calculated by using the most deviating vital signs and laboratory results from the Emergency Department or the first 24 h of Intensive Care Unit admission. All other variables in the APACHE-IV model remained similar

Both APACHE-IV models had an identical AUROC of 0.91 (95% CI 0.89–0.93) and 0.91 (95% CI 0.89–0.93). Calibration plots for both models were comparable (Additional file 5). The ED APACHE-IV score increased in 1075 (77.2%) patients, decreased in 33 (2.4%) patients, and remained unaltered in 284 (20.4%) patients. Predicted mortality increased ≥ 5.0% in 320 (23.1%) patients and decreased ≥ 5.0% in 1 (0.1%) patient (see Additional file 6). Higher ICU APACHE-IV scores had larger changes in predicted mortality risks if modified with ED variables.

### ED Length of stay and delta APACHE-IV score

**Fig. 2:**
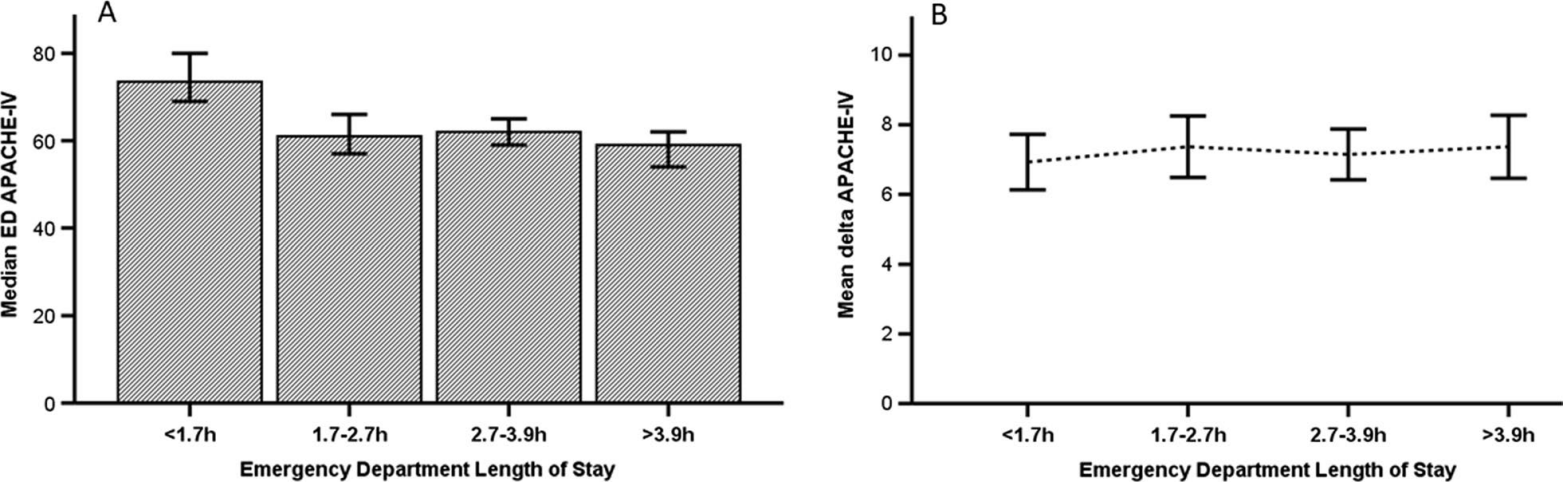
In panel A the median Emergency Department Acute Physiology and Chronic Health Evaluation (ED APACHE-IV) score is presented per quartile Emergency Department Length of Stay (ED-LOS), with 95% Confidence intervals. The ED APACHE-IV score uses the most deviated physiological variables from ED admission until 24 h after ICU admission, which differs from the ICU APACHE-IV score which only contains the most deviated physiological variables from the first 24 h of ICU admission. Panel B shows the mean delta APACHE-IV per quartile ED-LOS. The delta APACHE-IV is calculated as follows: ED APACHE-IV score—ICU APACHE-IV score

To study whether the ED LOS was associated with a delta APACHE-IV score (= ED APACHE-IV–ICU APACHE-IV), the ED APACHE-IV score and the delta APACHE-IV are presented per ED LOS quartile (Fig. 2). As assessed with a multivariable linear model, more fluid administration in the ED was associated with a significant adjusted increase in delta APACHE-IV (*p* value = 0.04) (see Table 3). Compared to those with an ED LOS < 1.7 h, among patients with ED LOS > 3.9 h the delta-APACHE was on average 1.6 points (95% CI 0.4–2.8) higher.

**Table 3:** Crude and adjusted associations between fluid administration, Emergency Department-Length of Stay and delta APACHE-IV

Independent variables	Crude Beta (95% CI)	P-value	Adjusted Beta (95% CI)	*p* value
No fluid	–	–	–	–
0-500 ml fluid	0.9 (− 1.0 to 1.2)	0.9	0.6 (− 0.6 to 1.8)	0.33
> 500 ml fluid	1.9 (1.0–2.8)	< 0.01	1.1 (0.0–2.1)	0.04
ED-lOS < 1.7 h	–	–	–	–
ED-LOS 1.7–2.7 h	− 0.4 (− 1.3 to 0.6)	0.45	0.8 (− 0.3 to 2.0)	0.15
ED-LOS 1.7–3.9 h	0.2 (− 0.7 to 1.2)	0.64	0.9 (− 0.2 to 2.1)	0.20
ED-LOS > 3.9 h	− 0.1 (− 1.0 to 0.9)	0.87	1.6 (0.4–2.8)	0.01

ED-LOS: Emergency Department Length of Stay, APACHE-IV: Acute Physiology and Chronic Health Evaluation (4^th^ edition), 95% CI, 95 percent confidence intervals

The association between fluid administration, ED-LOS and Delta APACHE-IV score (ED APACHE-IV–ICU APACHE-IV) was assessed. Crude Beta’s are presented, and adjusted Beta’s. Because of multicollinearity between ED LOS and fluid administration, separate models were used for both variables. Both models were adjusted for age, sex, hospital, and the modified APACHE-IV score

The median APACHE-IV score differed per quartile ED LOS (*p* < 0.01) calculated with the Kruskal Wallis Test.

## Discussion

The present study shows that in ICU patients admitted from the ED, the average calculated severity of illness and predicted mortality risks are substantially higher, and the SMR is lower if not only data from the first 24 h of ICU stay are used to calculate the APACHE-IV score, but also the data from the ED. In our population, the mean increase in APACHE IV score was 13%, with a mean increase in predicted mortality risk of 30% and a decrease in SMR of 13%.

Our results are in accordance with an earlier study of 76 ICU patients admitted from the ED and operating rooms [9]. In this study, higher APACHE II, APACHE III, and SAPS II scores were found if calculated with data from 6 h before ICU referral to 24 h after ICU admission compared with the standard period from ICU admission to 24 h after ICU admission. Consequently, including pre-ICU data leads to higher severity of illness scores. This can be explained by the fact that the period to choose the most deviating physiological variables becomes longer.

It may be argued that this influence of pre-ICU data on the assessment of severity of illness and predicted mortality has no major consequences, because the 24 h time period for data collection is clearly defined and similar for ICUs all over the world. Thus, APACHE scores and mortality predictions would be well comparable for describing case-mix and outcomes between ICUs (benchmarking). However, the true importance of our findings lies in the fact that major differences exist in the location where initial stabilization of patients takes place. For example, patients with septic shock are stabilized in the ED in some hospitals [16], whereas they would be transferred immediately to the ICU in other hospitals [8, 17, 18]. Especially in the early phase of stabilization, physiological parameters may be very disturbed leading to high APACHE scores. Because the APACHE prognostic scores are calculated from data exclusively from the first 24 h after ICU admission, this would contribute to higher APACHE scores and predicted mortality if stabilization took place in the ICU, but not if performed in the ED. Calculated severity of illness with the APACHE-IV score and predicted mortality risk will be lower in hospitals where patients are routinely stabilized in the ED before transfer to the ICU, compared to hospitals where patients are immediately transferred from the ED to the ICU and stabilized there [18]. As a result, the higher calculated SMRs may be falsely interpreted as a difference in the quality of care, while they can be fully explained by the fact that only data from the ICU are used for scoring. In our population, we found a substantial difference in SMR of 0.63 versus 0.55 depending on using or not using the ED data. In ICU literature this occurrence is referred to as lead-time bias [8, 9].

Our findings imply that whenever the quality of care among ICUs is compared using the SMR, a correction should be applied, or a margin of error of approximately 13% should be used if hospitals differ in their policy to stabilize patients. Our study was performed only in patients admitted to the ICU from the ED. However, in patients admitted from the operating rooms, wards, or in patients transferred from other hospitals, comparable differences in measured severity of illness and SMRs may be found if policies in stabilizing patients differ [8].

In the ICU APACHE-IV model, the calculated predicted mortality risk is already adjusted for the source of admission, e.g., from the ED. However, this adjustment does not correct for the influence of initial stabilization in either the emergency department or the ICU. In both situations, the patient is admitted from the ED with identical adjustment for source of admission. Future studies should investigate whether the APACHE-IV model could be improved to account for differences in hospital policy to stabilize patients.

We show that patients with higher ICU APACHE-IV scores had a larger absolute difference in predicted mortality compared to patients with a lower ICU APACHE-IV score. This may be explained by more intense resuscitative treatments in the ED. Unfortunately, the data available about therapies started in the ED were limited to only fluid therapy.

Small increases in delta APACHE-IV were found with more fluid administration in the ED and with an ED LOS of > 3.9 h. We can only speculate why a longer ED LOS led to a larger difference in APACHE-IV. Likely, patients with a prolonged ED LOS got more treatments in the ED, such as fluid administration, but also unregistered resuscitative therapies, which caused bigger changes in physiological signs than in patients who had a short ED LOS. However, we expected to find larger differences between the ICU APACHE-IV and ED APACHE-IV with more intensive ED treatment and a longer ED LOS. Nonetheless, this study analyzed a selective group of patients from the ED who may not have responded sufficiently to therapy and therefore may have been admitted to the ICU. This selection of ED patients may explain why we found only small increases in delta APACHE-IV in patients with a prolonged ED LOS.

Despite several strengths like the sample size and the multicenter design, our study also has some limitations. First, in approximately 18% of patients admitted from the ED to the ICU, no ED data were available. Patients could not be linked between databases for various reasons: i.e., transfers to other hospitals, patients who first went for surgery and not directly to the ICU, patients who bypassed the ED and went for coronary intervention, and possible registration problems. We cannot exclude that this may lead to some selection bias. Nonetheless, patient characteristics and mortality of patients who could not be linked between both databases were comparable with characteristics of included patients. Also, in our ED database, only initial values from ED admission were present. It may well be that even more deviating values could have been measured during ED treatment. Therefore, our findings likely underestimate the actual so-called lead-time bias in our patients. Furthermore, in our study, we did not include patients admitted to the ICU from an ED in another hospital. In those cases, the period between ED admission and ICU admission is prolonged, which potentially leads to more considerable differences in calculated APACHE IV scores and thus more lead-time bias [8].

## Conclusions

In summary, including the initial vital signs and laboratory results from the ED in the APACHE-IV score changed the predicted mortality risk and SMR, highlighting the influence of treatment given at the ED prior to IC admission. In addition, a longer ED LOS was associated with an increase in delta APACHE IV score, leading to a spuriously high SMR when this is not considered. Our findings are essential when comparing hospitals regarding quality of care and case-mix adjusted outcomes. APACHE IV scores and SMRs are not directly comparable in patients admitted from the ED if hospitals differ in their policy to stabilize patients in the ED before ICU admission. Future research should focus on refining models to adjust for these differences.

## Take home message

- The average calculated severity of illness and predicted mortality risks are substantially higher, and the SMR is 13% lower if not only data from the first 24 h of ICU stay are used to calculate the APACHE-IV score, but also the data from the ED.
- Predicted mortality risks and SMRs calculated by the APACHE IV scores are not directly comparable in patients admitted from the ED if hospitals differ in their policy to stabilize patients in the ED before ICU admission.

## Supplementary Information

**Additional file 1**. A table with variables is shown including the variables that are extracted from the NICE (Intensive Care database) and the NEED (The Emergency Department database) to calculate the Acute Physiology Score, as part of the Acute Physiology and Chronic Health Evaluation (APACHE)-IV.

**Additional file 2**. This file describes how points are given for each physiological parameter to calculate the Acute Physiology Score as part of the Acute Physiology and Chronic Health Evaluation (APACHE)-IV.

**Additional file 3**. Patient characteristics of excluded patients who could not be linked with the Netherlands Emergency department Database (N = 323).

**Additional file 4**. Additional patient characteristics including triage categories, top five presenting complaints, top seven reasons for admission to the ED, diagnostics in the ED and fluid administration.

**Additional file 5**. Calibration plots for both the ICU APACHE-IV score and the ED APACHE-IV score

**Additional file 6**. The change in predicted mortality for individual patients is presented if the ED Acute Physiology and Chronic Health Evaluation (APACHE)-IV is compared to the ICU APACHE-IV score.

## Data Availability

Data are available from the corresponding author upon reasonable request.

## Abbreviations

APACHE: Acute Physiology and Chronic Health Evaluation
AUROC: Area Under the Receiving Operating Characteristics
ED: Emergency Department
ICU: Intensive Care Unit
IQR: Interquartile Range
LOS: Length of Stay
NEED: The Netherlands Emergency department Evaluation Database
NICE: National Intensive Care Evaluation
SD: Standard deviation
SMR: Standardized Mortality Ratio
