# Correction: The effect of treatment and clinical course during Emergency Department stay on severity scoring and predicted mortality risk in Intensive Care patients

**DOI:** 10.1186/s13054-022-04008-x

**Published:** 2022-05-11

**Authors:** Bart G. J. Candel, Wouter Raven, Heleen Lameijer, Wendy A. M. H. Thijssen, Fabian Termorshuizen, Christiaan Boerma, Nicolette F. de Keizer, Evert de Jonge, Bas de Groot

**Affiliations:** 1 Department of Emergency Medicine, Leiden University Medical Centre, Albinusdreef 2, 2300 RC Leiden, The Netherlands; 2 Department of Emergency Medicine, Máxima Medical Centre, De Run 4600, 5504 DB Veldhoven, The Netherlands; 3 Department of Emergency Medicine, Medical Centre Leeuwarden, Henri Dunantweg 2, 8934 AD Leeuwarden, The Netherlands; 4 Department of Emergency Medicine, Catharina Hospital Eindhoven, Michelangelolaan 2, 5623 EJ Eindhoven, The Netherlands; 5 Department of Medical Informatics, Amsterdam University Medical Center, Meibergdreef 9, 1105 AZ Amsterdam, The Netherlands; 6 Amsterdam Public Health, Quality of Care, Amsterdam, The Netherlands; 7 National Intensive Care Evaluation (NICE) Foundation, Amsterdam, The Netherlands; 8 Department of Intensive Care, Medical Center Leeuwarden, Henri Dunantweg 2, 8934 AD Leeuwarden, The Netherlands; 9 Department of Intensive Care Medicine, Leiden University Medical Centre, Albinusdreef 2, 2300 RC Leiden, The Netherlands

## Correction to: Crit Care 26, 112 (2022). https://doi.org/10.1186/s13054-022-03986-2

Following publication of the original article [1], the authors identified an error in the author name Fabian Termorshuizen. The family name contained an error.

The incorrect author name is: Fabian Temorshuizen.

The correct author name is: Fabian Termorshuizen.

The author group has been updated above and the original article [1] has been corrected.

## References

[1] Candel BGJ, Raven W, Lameijer H (2022). The effect of treatment and clinical course during Emergency Department stay on severity scoring and predicted mortality risk in Intensive Care patients. Crit Care.

