# Supplementary material for: The effect of treatment and clinical course during Emergency Department stay on severity scoring and predicted mortality risk in Intensive Care patients
Source: Crit Care. 2022 Apr 19;26:112. doi: 10.1186/s13054-022-03986-2 (PMC9020059; doi:10.1186/s13054-022-03986-2)
Supplement: Supplementary file 1 — Additional file 1. A table with variables is shown including the variables that are extracted from the NICE (Intensive Care database) and the NEED (The Emergency Department database) to calculate the Acute Physiology Score, as part of the Acute Physiology and Chronic Health Evaluation (APACHE)-IV. [file 13054_2022_3986_MOESM1_ESM.docx]

**Supplemental digital content 1**

Table to calculate the Acute Physiology Score (APS) as part of the Acute Physiology and Chronic Health Evaluation (APACHE)-IV.

| **Variables** | **Registered in NICE** | **Registered in NEED** |
| --- | --- | --- |
| Age | Yes | Yes |
| *Acute Physiology Score (APS)* |  |  |
| - Heart rate | Yes | Yes |
| - Mean arterial pressure | Yes | Yes |
| - Temperature | Yes | Yes |
| - Respiratory Rate | Yes | Yes |
| - PaO2 (non-intubated patients, or intubated with FiO2<0.5) | Yes | Yes  patients were considered not intubated at ED arrival |
| - P(A-a)-O2 for intubated patients with FiO2 >0.5 | Yes | No |
| - PCO2 | Yes | Yes  If arterial PCO2 was not available, venous PCO2 was used with a correction of -4.8mmHg |
| - Hematocrit | Yes | Yes |
| - White blood cell count | Yes | Yes |
| - Creatinine | Yes | Yes |
| - Urine output | Yes | No |
| - Blood urea nitrogen | Yes | Yes |
| - Sodium | Yes | Yes |
| - Albumin | Yes | Yes |
| - Glucose | Yes | Yes |
| - Acid-base | Yes | Yes  If arterial pH was not available, venous pH was used with a correction of 0.03 |
| - Glasgow Coma Score (GCS) | Yes | Yes |
| *Chronic Health variables* |  |  |
| - AIDS | Yes | No |
| - Cirrhosis | Yes | No |
| - Hepatic failure | Yes | No |
| - Immunosuppression | Yes | No |
| - Lymphoma, leukemia, or myeloma | Yes | No |
| - Metastatic tumor | Yes | No |
| ICU admission diagnosis | Yes | No |
| ICU admission source (e.g., the ED) | Yes | No |
| Emergency surgery (Y/N) | Yes | No |
| Thrombolytic therapy for patients with myocardial infarction | Yes | No |
| Mechanical ventilation | Yes | No |
