# Supplementary material for: The effect of treatment and clinical course during Emergency Department stay on severity scoring and predicted mortality risk in Intensive Care patients
Source: Crit Care. 2022 Apr 19;26:112. doi: 10.1186/s13054-022-03986-2 (PMC9020059; doi:10.1186/s13054-022-03986-2)
Supplement: Supplementary file 2 — Additional file 2. This file describes how points are given for each physiological parameter to calculate the Acute Physiology Score as part of the Acute Physiology and Chronic Health Evaluation (APACHE)-IV. [file 13054_2022_3986_MOESM2_ESM.docx]

| **Supplemental digital content 2: Calculation of the Acute Physiology Score** | | | | | | |  |  |  |  |
| --- | --- | --- | --- | --- | --- | --- | --- | --- | --- | --- |
|  |  |  |  |  |  |  |  |  |  |  |
| In the left column the variables of the Acute Physiology Score as part of the APACHE-IV are given with their reference value. Points are assigned for values that are furthest away from this reference value. | | | | |  |  |  |  |  |  |
|  |  |  |  |  |  |  |  |  |  |  |
| **Pulse (beats/min)** | <=39 | 8 |  | Pulse points | 0 |  |  |  |  |  |
| *Select heart rate furthest from 75* | 40-49 | 5 |  |  |  |  |  |  |  |  |
|  | 50-99 | 0 |  |  |  |  |  |  |  |  |
|  | 100-109 | 1 |  |  |  |  |  |  |  |  |
|  | 110-119 | 5 |  |  |  |  |  |  |  |  |
|  | 120-139 | 7 |  |  |  |  |  |  |  |  |
|  | 140-154 | 13 |  |  |  |  |  |  |  |  |
|  | >=155 | 17 |  |  |  |  |  |  |  |  |
|  |  |  |  |  |  |  |  |  |  |  |
| **Mean Blood Pressure (MAP)** | <=39 | 23 |  | MAP points | 0 |  |  |  |  |  |
| *Select MAP furthest from 90* | 40-59 | 15 |  |  |  |  |  |  |  |  |
|  | 60-69 | 7 |  |  |  |  |  |  |  |  |
|  | 70-79 | 6 |  |  |  |  |  |  |  |  |
|  | 80-99 | 0 |  |  |  |  |  |  |  |  |
|  | 100-119 | 4 |  |  |  |  |  |  |  |  |
|  | 120-129 | 7 |  |  |  |  |  |  |  |  |
|  | 130-139 | 9 |  |  |  |  |  |  |  |  |
|  | >=140 | 10 |  |  |  |  |  |  |  |  |
|  |  |  |  |  |  |  |  |  |  |  |
| **Temperature (degrees Centigrade)** | <=32.9 | 20 |  | Temperature points | 0 |  |  |  |  |  |
| *Select core temperature furthest from 38.* | 33-33.4 | 16 |  |  |  |  |  |  |  |  |
| *Add 1 degree Centigrade to axillary temps* | 33.5-33.9 | 13 |  |  |  |  |  |  |  |  |
| *prior to selecting worst value* | 34-34.9 | 8 |  |  |  |  |  |  |  |  |
|  | 35-35.9 | 2 |  |  |  |  |  |  |  |  |
|  | 36-39.9 | 0 |  |  |  |  |  |  |  |  |
|  | >=40 | 4 |  |  |  |  |  |  |  |  |
|  |  |  |  |  |  |  |  |  |  |  |
| **Respiratory Rate (breaths/min)*** | <=5 | 17 |  | Respiratory rate points |  |  |  |  |  |  |
| *Select respiratory rate furthest from 19* | 6-11 | 8 |  |  |  |  |  |  |  |  |
| **for patients who are ventilated* | 12-13 | 7 |  |  |  |  |  |  |  |  |
| *no points are given for respiratory* | 14-24 | 0 |  |  |  |  |  |  |  |  |
| *rates of 6-12.* | 25-34 | 6 |  |  |  |  |  |  |  |  |
|  | 35-39 | 9 |  |  |  |  |  |  |  |  |
|  | 40-49 | 11 |  |  |  |  |  |  |  |  |
|  | >=50 | 18 |  |  |  |  |  |  |  |  |
|  |  |  |  |  |  |  |  |  |  |  |
| **PaO2 (mmHg)*** | <=49 | 15 |  | PaO2 points |  |  |  |  |  |  |
| **Use only for non-intubated patients or* | 50-69 | 5 |  |  |  |  |  |  |  |  |
| *intubated patients with FiO2 <0.5 (50%).* | 70-79 | 2 |  |  |  |  |  |  |  |  |
|  | >=80 | 0 |  | **OR** |  |  |  |  |  |  |
| **OR** |  |  |  |  |  |  |  |  |  |  |
|  |  |  |  |  |  |  |  |  |  |  |
| **A-aDO2*** | <100 | 0 |  | A-aDO2 points |  |  |  |  |  |  |
| **Only use A-aDO2 for intubated patients* | 100-249 | 7 |  |  |  |  |  |  |  |  |
| *with FiO2 >=0.5 (50%). Do not use PaO2* | 250-349 | 9 |  |  |  |  |  |  |  |  |
| *weights for these patients.* | 350-499 | 11 |  |  |  |  |  |  |  |  |
|  | >=500 | 14 |  |  |  |  |  |  |  |  |
|  |  |  |  |  |  |  |  |  |  |  |
| **Hematocrit (%)** | <=40.9 | 3 |  | Hematocrit points |  |  |  |  |  |  |
| *Select hematocrit furthest from 45.5.* | 41-49 | 0 |  |  |  |  |  |  |  |  |
|  | >=50 | 3 |  |  |  |  |  |  |  |  |
|  |  |  |  |  |  |  |  |  |  |  |
| **WBC (cu/mm)** | <1.0 | 19 |  | WBC points |  |  |  |  |  |  |
| *Select WBC furthest from 11.5.* | 1.0-2.9 | 5 |  |  |  |  |  |  |  |  |
|  | 3.0-19.9 | 0 |  |  |  |  |  |  |  |  |
|  | 20-24.9 | 1 |  |  |  |  |  |  |  |  |
|  | >=25 | 5 |  |  |  |  |  |  |  |  |
|  |  |  |  |  |  |  |  |  |  |  |
| **Creatinine without ARF* (mg/dl)** | <=0.4 | 3 |  | Creatinine without ARF points |  |  |  |  |  |  |
| *Select creatinine furthest from 1.0.* | 0.5-1.4 | 0 |  |  |  |  |  |  |  |  |
|  | 1.5-1.94 | 4 |  |  |  |  |  |  |  |  |
| **OR** | >=1.95 | 7 |  | **OR** |  |  |  |  |  |  |
|  |  |  |  |  |  |  |  |  |  |  |
| **Creatinine with ARF* (mg/dl)** | 0-1.4 | 0 |  | Creatinine with ARF points |  |  |  |  |  |  |
| **Acute Renal Failure (ARF) is defined as creatinine >=1.5 mg/dl* | >=1.5 | 10 |  |  |  |  |  |  |  |  |
| *as creatinine >=1.5 mg/dl* |  |  |  |  |  |  |  |  |  |  |
| *and urine output <410 cc/day and no* |  |  |  |  |  |  |  |  |  |  |
| *chronic dialysis* |  |  |  |  |  |  |  |  |  |  |
|  |  |  |  |  |  |  |  |  |  |  |
| **Urine Output (cc/day)** | <=399 | 15 |  | Urine output points |  |  |  |  |  |  |
| *Enter total for day* | 400-599 | 8 |  |  |  |  |  |  |  |  |
|  | 600-899 | 7 |  |  |  |  |  |  |  |  |
|  | 900-1499 | 5 |  |  |  |  |  |  |  |  |
|  | 1500-1999 | 4 |  |  |  |  |  |  |  |  |
|  | 2000-3999 | 0 |  |  |  |  |  |  |  |  |
|  | >=4000 | 1 |  |  |  |  |  |  |  |  |
|  |  |  |  |  |  |  |  |  |  |  |
| **BUN (mg/dl)** | <=16.9 | 0 |  | BUN points |  |  |  |  |  |  |
| *Select highest BUN (furthest from 0)* | 17-19 | 2 |  |  |  |  |  |  |  |  |
|  | 20-39 | 7 |  |  |  |  |  |  |  |  |
|  | 40-79 | 11 |  |  |  |  |  |  |  |  |
|  | >=80 | 12 |  |  |  |  |  |  |  |  |
|  |  |  |  |  |  |  |  |  |  |  |
| **Sodium (mEq/L)** | <=119 | 3 |  | Sodium points |  |  |  |  |  |  |
| *Select Sodium furthest from 145.5.* | 120-134 | 2 |  |  |  |  |  |  |  |  |
|  | 135-154 | 0 |  |  |  |  |  |  |  |  |
|  | >=155 | 4 |  |  |  |  |  |  |  |  |
|  |  |  |  |  |  |  |  |  |  |  |
| **Albumin (g/dl)** | <=1.9 | 11 |  | Albumin points |  |  |  |  |  |  |
| *Select albumin furthest from 3.5.* | 2.0-2.4 | 6 |  |  |  |  |  |  |  |  |
|  | 2.5-4.4 | 0 |  |  |  |  |  |  |  |  |
|  | >=4.5 | 4 |  |  |  |  |  |  |  |  |
|  |  |  |  |  |  |  |  |  |  |  |
| **Bilirubin (mg/dl)** | <=1.9 | 0 |  | Bilirubin points |  |  |  |  |  |  |
| *Select highest bilirubin (furthest from 0)* | 2.0-2.9 | 5 |  |  |  |  |  |  |  |  |
|  | 3.0-4.9 | 6 |  |  |  |  |  |  |  |  |
|  | 5.0-7.9 | 8 |  |  |  |  |  |  |  |  |
|  | >=8.0 | 16 |  |  |  |  |  |  |  |  |
|  |  |  |  |  |  |  |  |  |  |  |
| **Glucose (mg/dl)*** | <=39 | 8 |  | Glucose points |  |  |  |  |  |  |
| Select glucose furthest from 130 | 40-59 | 9 |  |  |  |  |  |  |  |  |
| **Glucose <=39 mg/dl is lower* | 60-199 | 0 |  |  |  |  |  |  |  |  |
| *weight than 40-59* | 200-349 | 3 |  |  |  |  |  |  |  |  |
|  | >=350 | 5 |  |  |  |  |  |  |  |  |
|  |  |  |  | Acid-Base points (see below) |  |  |  |  |  |  |
|  |  |  |  |  |  |  |  |  |  |  |
|  |  |  |  | GCS points (see below) |  |  |  |  |  |  |
|  |  |  |  |  |  |  |  |  |  |  |
|  |  |  |  | **TOTAL APS Score** | 0 |  |  |  |  |  |
|  |  |  |  |  |  |  |  |  |  |  |
| **Acid-Base Abnormalities*** |  | pCO2<25 | 25-<30 | 30-<35 | 35-<40 | 40-<45 | 45-<50 | 50-<55 | 55-<60 | >=60 |
| **Assign score based upon pH-pCO2* | pH <7.15 | 12 | | | | | | 4 | | |
| *relationship* | 7.15-<7.2 |  |  |  |  |  |  |  |  |  |
|  | 7.20-<7.25 | 9 | | 6 | | 3 | | 2 | | |
|  | 7.25-<7.30 |  |  |  |  |  |  |  |  |  |
|  | 7.30-<7.35 |  |  | 0 | | | 1 | | | |
|  | 7.35-<7.40 | 5 | |  |  |  |  |  |  |  |
|  | 7.40-<7.45 |  |  |  |  |  |  |  |  |  |
|  | 7.45-<7.50 |  |  | 0 | 2 | |  |  |  |  |
|  | 7.50-<7.55 |  |  |  |  |  |  |  |  |  |
|  | 7.55-<7.60 |  |  |  |  |  |  |  |  |  |
|  | 7.60-<7.65 | 0 |  | 3 |  |  |  | 12 |  |  |
|  | >=7.65 |  |  |  |  |  |  |  |  |  |
|  |  |  |  |  |  |  |  |  |  |  |
| **Neurological Abnormalities*** |  |  |  |  |  |  |  |  |  |  |
| **If a patient is anesthetized, under the influence of anesthesia, or totally paralyzed/sedated during the ENTIRE data collection period, attempt to obtain a GCS from the twelve-hour period prior to ICU admission when GCS was able to be assessed. If no assessable GCS is documented during that time period, zero points shhould be assigned. If unable to determine verbal score (due to intubation status or similar barriers), use clinical judgement and assign Glasgow Verbal Score according to the following scale:* | | | | |  |  |  |  |  |  |
|  |  |  |  |  |  |  |  |  |  |  |
|  |  |  |  |  |  |  |  |  |  |  |
|  |  |  |  |  |  |  |  |  |  |  |
| *alert, oriented 5* |  |  |  |  |  |  |  |  |  |  |
| *confused 3* |  |  |  |  |  |  |  |  |  |  |
| *nonresponsive 1* |  |  |  |  |  |  |  |  |  |  |
|  |  |  |  |  |  |  |  |  |  |  |
| ***If patient's eyes open spontaneously (4) or to*** | verbal | oriented, converses (5) | | confused conversation (4) | | inappropriate words, incomp. sounds (3,2) | | no response (1) | |  |
| ***painful/verbal stimulation (2,3)****, use scale:* | motor |  |  |  |  |  |  |  |  |  |
| *Note: shaded areas represent unlikely clinical combinations. Placing a patient in any of these cells should be done after careful confirmation of clinical findings.* | obeys verbal commands (6) | 0 | | 3 | | 10 | | 15 | |  |
|  | localizes pain (5) | 3 | | 8 | | 13 | | 15 | |  |
|  | flexion withdrawal/decorticate rigidity (4,3) | 3 | | 13 | | 24 | | 24 | |  |
|  | decerebrate rigidity/no response (2,1) | 3 | | 13 | | 29 | | 29 | |  |
|  |  |  |  |  |  |  |  |  |  |  |
| ***If patient's eyes do not open spontaneously*** | verbal | oriented, converses (5) | | confused conversation (4) | | inappropriate words, incomp. sounds (3,2) | | no response (1) | |  |
| ***or to painful/verbal stimulation (1)****, use scale:* | motor |  |  |  |  |  |  |  |  |  |
| *Note: shaded areas represent extremely unlikely clinical combinations, and should not be used. If these combinations are verified in a clinical setting, no prediction should be generated for the patient.* | obeys verbal commands (6) |  | |  | |  | | 16 | |  |
|  | localizes pain (5) |  | |  | |  | | 16 | |  |
|  | flexion withdrawal/decorticate rigidity (4,3) |  | |  | | 24 | | 33 | |  |
|  | decerebrate rigidity/no response (1) |  | |  | | 29 | | 48 | |  |
|  |  |  |  |  |  |  |  |  |  |  |
|  |  |  |  |  |  |  |  |  |  |  |
|  |  |  |  |  |  |  |  |  |  |  |
|  |  |  |  |  |  |  |  |  |  |  |
|  |  |  |  |  |  |  |  |  |  |  |
|  |  |  |  |  |  |  |  |  |  |  |
|  |  |  |  |  |  |  |  |  |  |  |
|  |  |  |  |  |  |  |  |  |  |  |
|  |  |  |  |  |  |  |  |  |  |  |
|  |  |  |  |  |  |  |  |  |  |  |
|  |  |  |  |  |  |  |  |  |  |  |
