# Supplementary material for: The effect of treatment and clinical course during Emergency Department stay on severity scoring and predicted mortality risk in Intensive Care patients
Source: Crit Care. 2022 Apr 19;26:112. doi: 10.1186/s13054-022-03986-2 (PMC9020059; doi:10.1186/s13054-022-03986-2)
Supplement: Supplementary file 3 — Additional file 3. Patient characteristics of excluded patients who could not be linked with the Netherlands Emergency department Database (N = 323). [file 13054_2022_3986_MOESM3_ESM.docx]

**Supplementary file 3**

Patient characteristics of excluded patients who could not be linked with the Emergency Department Database (N=323)

| **Characteristics**  **(N=323)** | **Intensive Care Unit** | **Intensive Care Unit** |
| --- | --- | --- |
| **Age, years, median (IQR)** | 65 (53-74) | - |
| **Sex, male, N (%)** | 221 (68.4) | - |
| **In hospital mortality, N (%)** | 55 (17.0) |  |
| **Vital signs, median (IQR) {missing}** | **Lowest values <24h** | **Highest values <24h** |
| MAP, mmHg | 63 (53-72) {2} | 99 (88-116) {3} |
| HR, bpm | 71 (59-83) {3} | 103 (88-119) {1} |
| RR, /min. | 12 (10-15) {4} | 24 (22-27) {5} |
| Temperature, ᵒC | 36.3 (35.3-36.9) {2} | 37.4 (36.7-37.9) {4) |
| GCS | 15 (6-15) |  |
| Urine, 24hours, L | 1.2 (0.8-1.9) {28} |  |
| Creatinine, µmol/L | 82 (66-108) {26} | 91 (71-120) {27} |
| Urea, mmol/L | 7.0 (5.6-9.7) {30} |  |
| Hematocrit, L/L | 0.36 (0.32-0.41) {12} | 0.41 (0.35-0.45) {13} |
| Leukocytes, x10^9/L | 10.9 (8.1-14.4) {54} | 12.1 (9.2-16.9) {55} |
| Sodium, mmol/L | 137 (135-139) {17} | 140 (138-142) {18} |
| Albumin, g/L | 29 (25-34) {161} | 30 (25-34) {161) |
| Glucose, mmol/L | 6.2 (5.4-7.1) {18} | 9.1 (7.0-11.8) {19} |
| Bilirubin, µmol/L | 10 (7-16) {149} |  |
| a-PO2, mmHg | 88 (73-107) {103} |  |
| a-PCO2, mmHg | 38 (35-44) {103} |  |
| a-pH | 7.40 (7.30-7.40) {103} |  |
| **Mechanical ventilation, N (%)** | - | 170 (52.6) |
| FiO2 (%), median (IQR) | - | 40 (30-50) {90} |

ICU: Intensive Care Unit, IQR: Interquartile Range, N: number, MAP: mean arterial pressure, mmHg: millimeter mercury, HR: heart rate, SpO2: peripheral oxygen saturation, RR: respiratory rate, ᵒC: degrees Celsius, GCS: Glasgow Coma Scale, L: Liter, a-PO2: arterial partial pressure of oxygen, a/v-PCO2: arterial or venous partial pressure of carbon dioxide, a/v-pH: arterial acid base, FiO2: Fraction of inspired oxygen.

From the ICU, both the lowest and highest values are given from the first 24hours after admission, if available.
