# Supplementary material for: The effect of treatment and clinical course during Emergency Department stay on severity scoring and predicted mortality risk in Intensive Care patients
Source: Crit Care. 2022 Apr 19;26:112. doi: 10.1186/s13054-022-03986-2 (PMC9020059; doi:10.1186/s13054-022-03986-2)
Supplement: Supplementary file 4 — Additional file 4. Additional patient characteristics including triage categories, top five presenting complaints, top seven reasons for admission to the ED, diagnostics in the ED and fluid administration. [file 13054_2022_3986_MOESM4_ESM.docx]

| **Demographics** | **N = 1398** |
| --- | --- |
| **Arrived by ambulance, N (%)** | 985 (81.4) {188} |
| **Triage category in the ED, N (%)** | {16} |
| Green/blue (non-urgent) | 67 (5.0) |
| Yellow (less urgent) | 340 (25.2) |
| Orange (urgent) | 636 (47.2) |
| Red (Immediate) | 305 (22.6) |
| **Top five chief complaints in the ED, N (%)** | {246} |
| Feeling unwell | 222 (15.9) |
| Dyspnea | 216 (15.5) |
| Intoxication | 154 (11.0) |
| Abdominal pain | 130 (9.3) |
| Thoracic pain | 84 (6.0) |
| Miscellaneous | 592 (42.3) |
| **Top seven reasons for ICU admission, N (%)** |  |
| Drug overdose | 205 (14.7) |
| Sepsis | 188 (13.4) |
| After cardiac arrest | 136 (9.7) |
| Peripheral vascular surgery -surgical | 87 (6.2) |
| Infection | 82 (5.9) |
| Pulmonary embolism | 80 (5.7) |
| Cardiovascular | 75 (5.4) |
| Miscellaneous | 545 (39.0) |
| **Diagnostics in the ED, N (%)** |  |
| Blood cultures | 335 (19.4) |
| ECG | 992 (57.3) |
| Radiology | 962 (68.8) |
| **Fluid administration in the ED** |  |
| 0 ml | 1085 (62.7) |
| 0-500ml | 241 (13.9) |
| >500ml | 404 (23.4) |
|  |  |

**Supplemental digital content 4** Patient demographics including triage categories, top five presenting complaints, top seven reasons for admission to the ED, diagnostics in the ED and fluid administration.

ED: Emergency Department, ECG: Electrocardiogram

Numbers between {} represent missings.

Triage categories are based on the Manchester Triage System or the Dutch Triage Standard.
