# Supplementary material for: The effect of treatment and clinical course during Emergency Department stay on severity scoring and predicted mortality risk in Intensive Care patients
Source: Crit Care. 2022 Apr 19;26:112. doi: 10.1186/s13054-022-03986-2 (PMC9020059; doi:10.1186/s13054-022-03986-2)
Supplement: Supplementary file 5 — Additional file 5. Calibration plots for both the ICU APACHE-IV score and the ED APACHE-IV score [file 13054_2022_3986_MOESM5_ESM.pptx]

## Slide 1
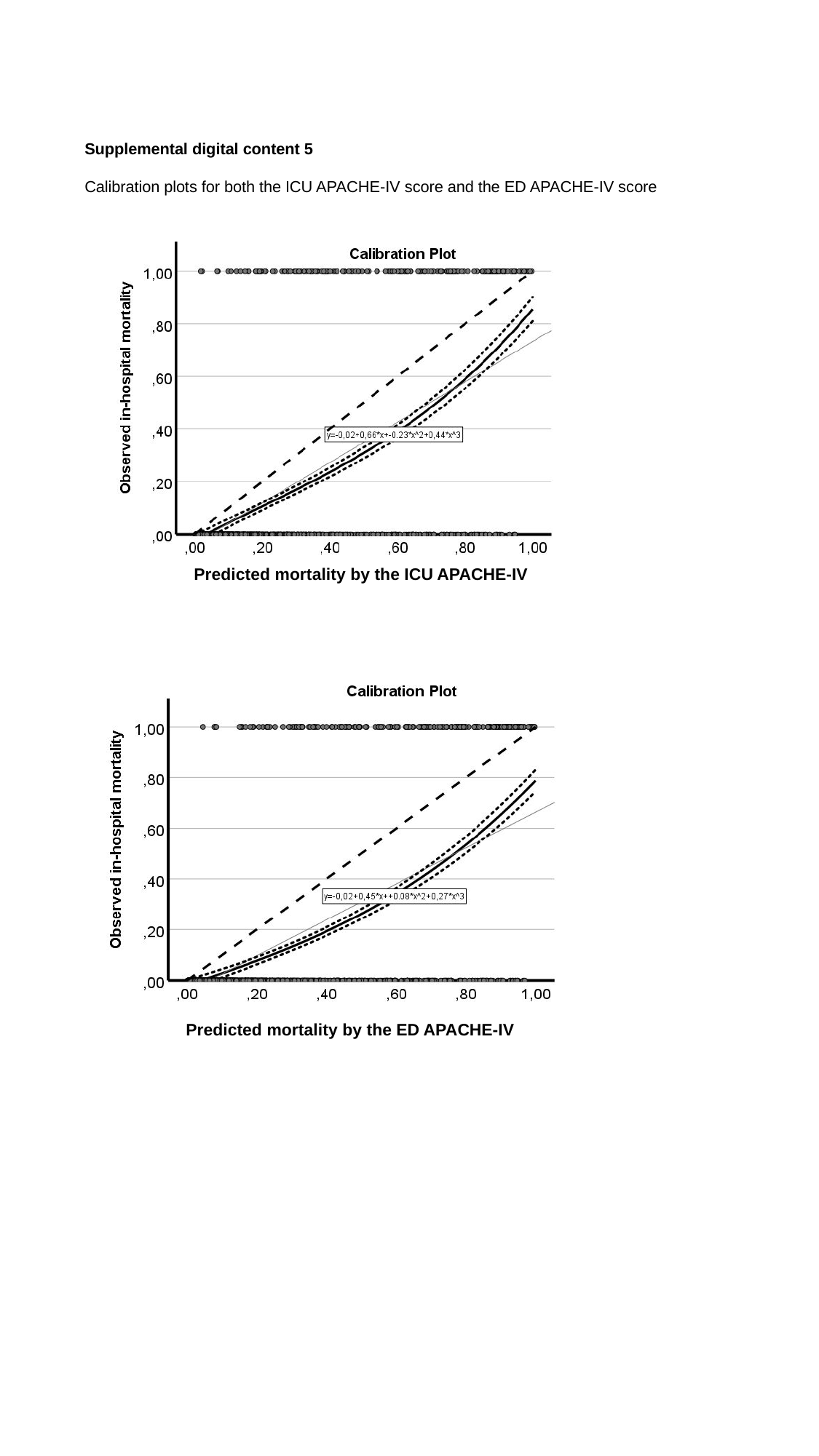

Supplemental digital content 5
Calibration plots for both the ICU APACHE-IV score and the ED APACHE-IV score
Predicted mortality by the ICU APACHE-IV
Predicted mortality by the ED APACHE-IV
