# Supplementary material for: The effect of treatment and clinical course during Emergency Department stay on severity scoring and predicted mortality risk in Intensive Care patients
Source: Crit Care. 2022 Apr 19;26:112. doi: 10.1186/s13054-022-03986-2 (PMC9020059; doi:10.1186/s13054-022-03986-2)
Supplement: Supplementary file 6 — Additional file 6. The change in predicted mortality for individual patients is presented if the ED Acute Physiology and Chronic Health Evaluation (APACHE)-IV is compared to the ICU APACHE-IV score. [file 13054_2022_3986_MOESM6_ESM.pptx]

## Slide 1
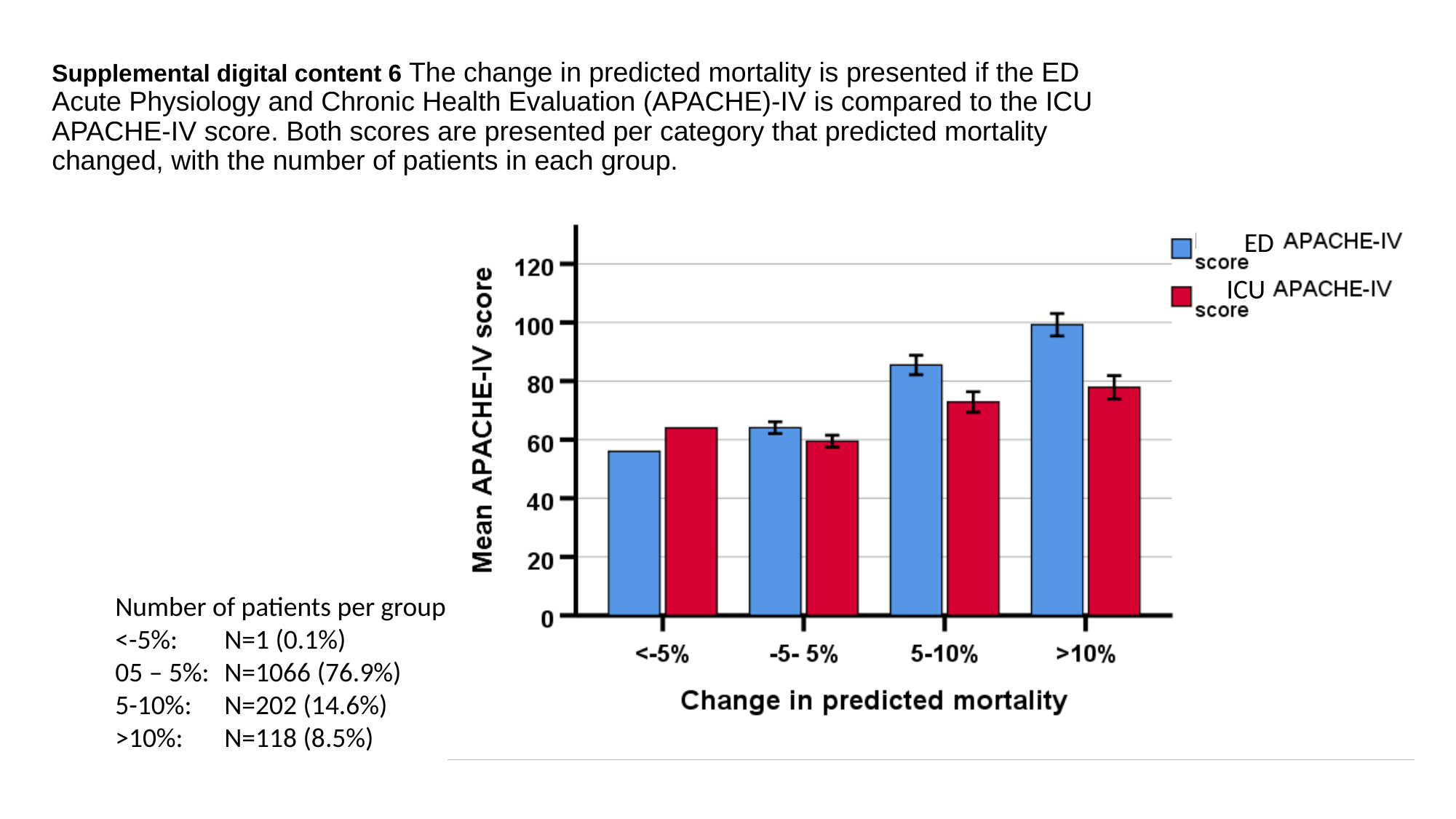

Supplemental digital content 6 The change in predicted mortality is presented if the ED Acute Physiology and Chronic Health Evaluation (APACHE)-IV is compared to the ICU APACHE-IV score. Both scores are presented per category that predicted mortality changed, with the number of patients in each group.
ED
ICU
Number of patients per group:
<-5%: 	N=1 (0.1%)
05 – 5%: 	N=1066 (76.9%)
5-10%:	N=202 (14.6%)
>10%:	N=118 (8.5%)
